# Commentary: Stress Signal Network between Hypoxia and ER Stress in Chronic Kidney Disease

**DOI:** 10.3389/fphys.2017.00243

**Published:** 2017-04-25

**Authors:** Priscila F. Tempaku, Sergio Tufik, Camila Hirotsu

**Affiliations:** Department of Psychobiology, Universidade Federal de São PauloSão Paulo, Brazil

**Keywords:** chronic kidney disease, hypoxia, er stress, sleep, obstructive sleep apnea

## To the editor

Recently, Maekawa and Inagi interestingly reported that hypoxia and endoplasmic reticulum (ER) stress might be associated with chronic kidney disease (CKD) in the review article entitled “Stress signal network between hypoxia and ER stress in chronic kidney disease” (Maekawa and Inagi, 2017). We would like to congratulate the authors for their elegant work and to stress the great implication of these findings since CKD is one of the most prevalent incurable diseases worldwide that considerably affects the patient's life. Notwithstanding the mechanisms presented by Maekawa and Inagi, we would like to highlight the potential contributing role of obstructive sleep apnea (OSA) in the association found between hypoxia and ER stress with the deterioration of kidney function.

OSA is the most prevalent sleep-disordered breathing, occurring in up to one third of the general population (Tufik et al., 2010; Heinzer et al., 2015). It is characterized by increased upper airway resistance and intermittent breathing pauses during sleep, leading to oxygen desaturation and sleep fragmentation (Epstein et al., 2009). Due mainly to intermittent hypoxia-related mechanisms, OSA has been associated with increased oxidative stress (Lavie, 2015) and inflammation (Zhang et al., 2014), which in turn may become potentially harmful to various tissues and organs (Anandam et al., 2013; Salord et al., 2014).

Cumulative evidence suggests that the association between OSA and CKD is bidirectional (Abuyassin et al., 2015). It is estimated that the prevalence of OSA in CKD is about 10 times higher compared to the prevalence of OSA in the general population (Hanly, 2004). On the other hand, a positive correlation between OSA severity and renal function impairment has also been reported, demonstrating that OSA may be an important independent risk factor for initiation and progression of CKD (Chou et al., 2011). The proposed mechanisms that relate OSA to the deterioration of kidney function, thus leading to CKD, involve nocturnal hypoxemia and activation of renin-angiotensin system that occurs in response to oxidative stress (Ahmed et al., 2011; Zalucky et al., 2015).

As mentioned by Maekawa and Inagi (2017), renal hypoxia is a meaningful factor for the development of CKD. Hypoxemia, a condition associated with OSA, represents a common and direct pathway to the development of renal tissue hypoxia. A recent study in an animal model of OSA using chronic intermittent hypoxia (CIH) showed that CIH accelerated renal histological injury due to upregulation of the advanced glycation end products (RAGE) receptor and its ligand high mobility group box 1 (HMGB1), which are both increased in several renal disorders. RAGE and HMGB1 activated chronic inflammatory transduction cascades, amplifying the inflammatory response through the recruitment of cytokines and effector molecules, which contributes to nephron apoptosis and accelerated renal dysfunction (Wu et al., 2016). Additionally, OSA can disturb the homeostasis of ER through the long-term exposure to intermittent cycles of hypoxia-reoxygenation. Many motor neurons process large amounts of proteins that must be properly folded within the ER, thus they are prone to experiencing ER stress and the unfolded protein response (UPR). Short-term intermittent hypoxia selectively activates the protein kinase RNA-like endoplasmic reticulum kinase (PERK) pathway of the UPR in some motor nuclei including the hypoglossal and facial. After 8 weeks of CIH, mice presented a similar PERK activation in both the hypoglossal and facial motor neurons, which were associated with increased UPR (Zhu et al., 2008). The inability of these neurons to relieve the ER stress leads to a neural injury that is observed at the ultrastructural level (Zhu et al., 2008). However, the role of OSA-induced hypoxia in systemic ER stress is still unknown.

Other mechanisms that potentially relate OSA to CKD development is the activation of the renin-angiotensin system (Zalucky et al., 2015). This system plays an essential role in the maintenance of renal hemodynamics, as well as in the regulation of renal sodium transport, glomerular hemodynamics, and glomerular filtration in both physiological and pathological conditions (Navar, 2014). Acute hypoxia leads to stimulation of the peripheral chemoreceptors, which increase sympathetic outflow, thus elevating acutely the blood pressure. However, in long-term, these repeated episodic or intermittent periods of hypoxia activates the renin-angiotensin system (Fletcher, 2001). Evidence has shown that the nocturnal hypoxemia influences the renal renin-angiotensin system activation, being detrimental to negative vascular and renal outcomes (Zalucky et al., 2015). Of note, a continuous positive airway pressure (CPAP) intervention, which is the gold-standard treatment for OSA, seems to improve renal hemodynamics and to down-regulate the renal renin-angiotensin system activity (Nicholl et al., 2014).

Finally, considering the current scenario of evidence, it is important to understand whether ER stress plays a role in the mechanisms by which OSA has been associated with kidney function reduction and a higher risk of CKD so that other therapeutic and complimentary strategies start to develop in order to prevent kidney failure in OSA patients.

## Author contributions

PFT has worked with the literature research, the content of the text, references, and overall manuscript's structure. CH has contributed with the conception and design of the work, manuscript preparation, and the review of the files. ST has revised the manuscript.

## Funding

Our work has been supported by grants from the Associação Fundo de Incentivo à Pesquisa (AFIP), São Paulo Research Foundation (FAPESP, #2014/15259-2 to CH and #2015/17549-0 to PFT), and National Council for Scientific and Technological Development (CNPq).

### Conflict of interest statement

The authors declare that the research was conducted in the absence of any commercial or financial relationships that could be construed as a potential conflict of interest.
